# Highly efficient separation and enrichment of polyphenols by 6-aminopyridine-3-boronic acid-functionalized magnetic nanoparticles assisted by polyethylenimine

**DOI:** 10.1039/d1ra08751g

**Published:** 2022-03-01

**Authors:** Yansong Zhang, Lianglei Qing, Linna Xu

**Affiliations:** College of Food and Drug, Luoyang Normal University Luoyang 471934 China nora_zhang@163.com

## Abstract

Polyphenols have found a lot of therapeutic effects and potential applications such as antioxidant, anti-inflammatory, mutant resistance, immunosuppressant and anti-tumor properties. They can be divided into five main classes, namely flavonoids, phenolic acids, stilbenes, lignans, and others. Thus, the content detection of polyphenols in real samples such as fruit juice and tea is of great significance. Due to the presence of complex interfering components in actual samples, separation and enrichment of polyphenols prior to analysis is key. Therefore, it is quite necessary to establish a simple, low-cost and efficient purification method for *cis*-diol-containing polyphenols from real samples. Boronate affinity materials are able to reversibly bind *cis*-diol-containing compounds by forming a five- or six-membered boronic cyclic ester in aqueous media. However, conventional boronate affinity materials exhibited low binding capacity and high binding pH. In this study, the polyethyleneimine (PEI)-assisted 6-aminopyridine-3-boronic acid functionalized magnetic nanoparticles (MNPs) were developed to capture efficiently *cis*-diol-containing polyphenols under neutral condition. PEI was applied as a scaffold to amplify the number of boronic acid moieties. While 6-aminopyridine-3-boronic acid was used as an affinity ligand due to low p*K*_a_ value and excellent water solubility toward polyphenols. The results indicated that the prepared boronic acid-functionalized MNPs provided high binding capacity and fast binding kinetics under neutral conditions. In addition, the obtained MNPs exhibited relatively high binding affinity (*K*_d_ ≈ 10^−4^ M), low binding pH (pH ≥ 6.0) and tolerance of the interference of abundant sugars.

## Abstract 2

Synthesis routes of 6-aminopyridine-3-boronic acid-functionalized magnetic nanoparticles assisted by polyethylenimine (Fe_3_O_4_@PEI@PYBA).

## Graphical abstract

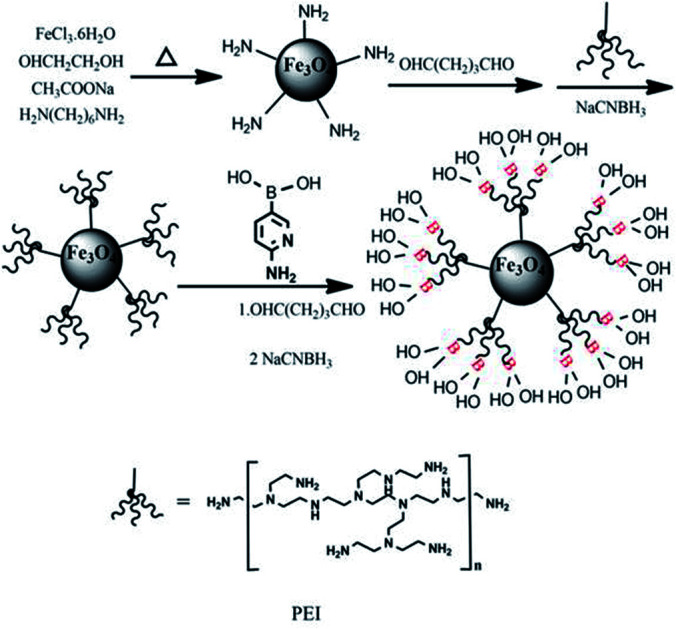

## Introduction

Polyphenols are a class of important compounds naturally present in plants including vegetables, fruits and teas. And they are gaining more and more attention due to their therapeutic effects such as antioxidant, anti-inflammatory, mutant resistance, immunosuppressant and anti-tumor properties.^1^ In addition, polyphenolic compounds also can be applied in many terminal products such as food dyes, bioactive packaging, paints, fertilizers, surfactants, rubber, plastics and curing agents.^2^ Polyphenols have aromatic rings with two or more hydroxyl groups. They can be divided into five main classes, namely flavonoids, phenolic acids, stilbenes, lignans, and others.^3,4^ Among them, flavonoids are the most abundant compounds because they are mainly synthesized through the phenylpropanoid pathway in plants.^5^ As derivatives of benzoic acid and cinnamic acid, phenolic acids are characterized by a high antioxidant activity.^6^ In addition, stilbenes are present in low abundance in the human diet in foods such as grapes, berries, peanuts, red wine, *etc.*^7^ The lignans are formed by two units of a phenylpropane derivative and found in wheat, citrus fruits, onions, *etc.* Therefore, the content detection of the above mentioned polyphenols, especially in fruit juice, beverage, tea, and Chinese medicine related drinks, is of great significance. Due to the complex interfering components in actual sample, separation and enrichment of polyphenols prior to analysis is of vital importance.^8^

Fortunately, most polyphenols contain *cis*-diol structure.^9^ Thus, they can be selectively separated and enriched by boronic acid-functionalized MNPs. Boronic acid-functionalized materials have been widely applied for selective extraction and separation of *cis*-diol-containing compounds.^10–36^ However, the binding capacity of conventional boronic acids functionalized materials toward *cis*-diols is relatively low. The number of boronic acid sites can be amplified using highly branched polyethyleneimine (PEI). PEI has several advantages including flexible chains, hydrophilic properties, easy post-modification, plentiful amino groups and low cost.^37^ Therefore, the PEI-assisted boronic acid functionalized MNPs are able to provide higher binding capacity. In addition, the use of alkaline solution exhibits two disadvantages. First, it is easy to oxidize the *cis*-diols of polyphenols.^38^ Second, the discharge of alkaline solution may cause secondary pollution in water. To this end, Li and co-workers prepared PEI-assisted 3-carboxybenzoboroxole functionalized MNPs for the specific capture of *cis*-diol-containing flavonoids under neutral conditions.^39^ However, due to its complex and difficult preparation, the boronic acid ligand 3-carboxybenzoboroxole is not suitable for industrial production. Furthermore, 3-carboxybenzoboroxole is not especially advantageous to acidic pH conditions for high binding capacity. Compared with the boronic acid ligand 3-carboxybenzoboroxole, 6-aminopyridine-3-boronic acid is more advantageous to acidic pH conditions due to the presence of a nitrogen atom in the heterocyclic ring. And 3-carboxybenzoboroxole require difficult preparation while 6-aminopyridine-3-boronic acid is commercially available.^40^ Thus, it is necessary to develop PEI-assisted 6-aminopyridine-3-boronic acid functionalized MNPs in order to address the issues of 3-carboxybenzoboroxole. Although 6-aminopyridine-3-boronic acid had been used as affinity ligands to prepare boronic acid functionalized MNPs,^40^ PEI was not applied in this reference, which would affect the binding properties to some extent. In addition, this is first report on boronic acid functionalized materials using polyphenols as the research targets.

In the study, we applied 6-aminopyridine-3-boronic acid and PEI as affinity ligands and a scaffold to prepare PEI-assisted 6-aminopyridine-3-boronic acid-functionalized MNPs for highly efficient capture of *cis*-diol-containing polyphenols under neutral condition. In this work, MNPs were used as supporting nanomaterials due to their good biocompatibility, superparamagnetic property, low toxicity and easy preparation.^30,41^ The preparation process includes the following several steps. First, NH_2_-functionalized MNPs were synthesized and then PEI was modified to MNPs through Schiff base reaction. Subsequently, PEI modified MNPs were functionalized with 6-aminopyridine-3-boronic acid. Due to the combination of PEI with 6-aminopyridine-3-boronic acid, the prepared boronic acid functionalized MNPs exhibited relatively high binding affinity (*K*_d_ ≈ 10^−4^ M), low binding pH (pH ≥ 6.0) and fast binding kinetics under neutral condition. In addition, the obtained boronate affinity MNPs exhibited high binding capacity and tolerance of the interference to abundant sugars.

## Experimental

### Reagents and materials

Branched polyethyleneimine (PEI) (*M*_w_ = 600, 1800, 10 000 and 70 000), 6-aminopyridine-3-boronic acid (PYBA), adenosine, deoxyadenosine, caffeic acid (CA), quercetin (QUE), catechin (CAT), piceatannol (PIC), *p*-hydroxy-cinnamic acid (PCA), kaempferol (KAE), epiafzelechin (EPI), resveratrol (RES) were obtained from J&K scientific (Shanghai, China). FeCl_3_·6H_2_O, NaOAc, ethylene glycol, sodium cyanoborohydride, anhydrous methanol and glutaraldehyde were purchased from Alfa Aesar (Tianjin, China). All reagents were of analytical grade.

### Instruments

Transmission electron microscopy (TEM) was carried out on a JEM-1010 system (JEOL, Japan). UV absorbance data were measured on a U-3010 UV spectrophotometer (Kyoto, Japan).

### Preparation of Fe_3_O_4_@PEI@PYBA

**Fig. 1:**
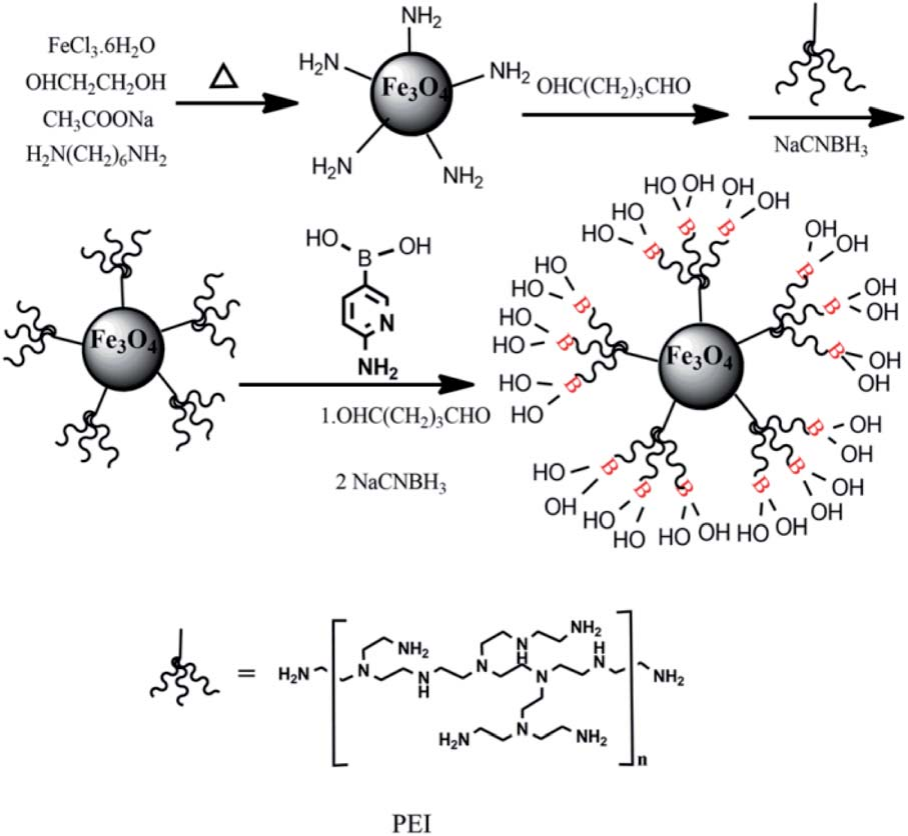
Synthesis routes of Fe_3_O_4_@PEI@PYBA.

As depicted in Fig. 1, Fe_3_O_4_@PEI@PYBA was prepared according to the below three steps: (1) synthesis of NH_2_-functionalized MNPs, (2) preparation of PEI modified MNPs (Fe_3_O_4_@PEI) *via* Schiff base reaction of glutaraldehyde, (3) functionalization of 6-aminopyridine-3-boronic acid by the Schiff base reaction of glutaraldehyde. The NH_2_-functionalized MNPs were synthesized according to a previously reported method.^42^ The above NH_2_-functionalized MNPs of 200 mg were added to 40 mL anhydrous methanol containing 10% glutaraldehyde, and the mixture was mechanically stirred for 4 h at room temperature. The glutaraldehyde-activated MNPs were washed three times with anhydrous methanol and then dispersed in 30 mL anhydrous methanol containing 0.8 g PEI by ultrasound. 1% (w/w) sodium cyanoborohydride was added (200 mg every 6 hours) during the course of reaction. The Fe_3_O_4_@PEI were collected by a magnet, washed with water and ethanol for 3 times each, and then dried at 40 °C overnight.

In addition, the Fe_3_O_4_@PEI of 100 mg was added to 20 mL anhydrous methanol containing 10% glutaraldehyde and the mixture was stirred for 4 h at room temperature. Then, the obtained glutaraldehyde-activated NPs were dispersed in 30 mL anhydrous methanol containing 0.6 g PYBA. Finally, cyanoborohydride was added into the above solution (200 mg every 6 hours) for 24 h. The obtained Fe_3_O_4_@PEI@PYBA was separated from reaction mixtures using a magnet, and was washed with water and ethanol and dried at 50 °C. The obtained Fe_3_O_4_@PEI@PYBA was stored for further use.

### Selectivity of Fe_3_O_4_@PEI@PYBA

The selectivity of Fe_3_O_4_@PEI@PYBA was evaluated using caffeic acid (CA), quercetin (QUE), catechin (CAT) and piceatannol (PIC) as *cis*-diol-containing polyphenolic samples while *p*-hydroxy-cinnamic acid (PCA), kaempferol (KAE), epiafzelechin (EPI), resveratrol (RES) were used as non-*cis*-diol analogue. First, the above mentioned each polyphenol solution of 0.5 mg mL^−1^ was prepared with 50 mM phosphate buffer (pH 7.0). Then equivalent Fe_3_O_4_@PEI@PYBA (5 mg) were added to 500 μL of the polyphenol solutions in centrifugal tubes, respectively. Then, they were shaken on a rotator at room temperature for 2 h. The Fe_3_O_4_@PEI@PYBA was then collected and rinsed with 500 μL PBS (pH 7.0) for three times. After washing, the MNPs were eluted in 100 μL acetic acid solution for 1 h on a rotator and the eluates were collected. The eluent containing these polyphenols adsorbed by the Fe_3_O_4_@PEI@PYBA were measured with UV-vis spectrophotometer. UV absorbance was adopted at 350 nm, 376 nm, 280 nm, 330 nm, 300 nm, 365 nm, 285 nm and 308 nm for CA, QUE, CAT, PIC, PCA, KAE, EPI and RES, respectively. UV absorbance measurement was repeated three times.

### Kinetic adsorption experiment

CA was applied as a representative sample to investigate the kinetic adsorption experiment. Fe_3_O_4_@PEI@PYBA of 3 mg was added to 0.5 mL of CA solution (1 mg mL^−1^) and the obtained suspension was shaken for different time from 1 to 14 min at room temperature. The obtained suspension at different interaction time were collected by magnetic force and rinsed with PBS (pH 7.0) for three times. After washing, CA bound on Fe_3_O_4_@PEI@PYBA was eluted with acetic acid solutions (pH 2.7) and the supernatant was measured by absorbance analysis.

### Measurement of adsorption isotherm and Scatchard analysis

The measurement of adsorption isotherm and Scatchard analysis was carried out according to the following process. First, an amount of Fe_3_O_4_@PEI@PYBA was dispersed to 50 mM PBS to form homogeneous suspension (20 mg mL^−1^). Then, 300 μL solutions at different concentrations of CA were added to the above Fe_3_O_4_@PEI@PYBA suspension of 200 μL and shaken on a rotator for 1 h at room temperature. Thereafter, CA-bound Fe_3_O_4_@PEI@PYBA were separated and then washed with PBS (pH 7.0) for 3 times. Finally, the compounds bound on the Fe_3_O_4_@PEI@PYBA were eluted by acetic acid solution. The eluent containing CA was measured with absorbance.

The Scatchard analysis was performed according to a previously reported method.^43^ Dissociation constant (*K*_d_) and binding capacity (*Q*_max_) were evaluated based on the following Scatchard equation:
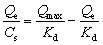
where *Q*_max_ and *K*_d_ is the saturated binding capacity and the dissociation constant, respectively, *Q*_e_ is the binding amount of CA to Fe_3_O_4_@PEI@PYBA at equilibrium, *C*_s_ is the free concentration at binding equilibrium. The values for *K*_d_ and *Q*_max_ are evaluated based on the slope and the intercept of plots of *Q*_e_/*C*_s_*versus Q*_e_.

## Results and discussion

### Characterization of the magnetic nanoparticles (MNPs)

**Fig. 2:**
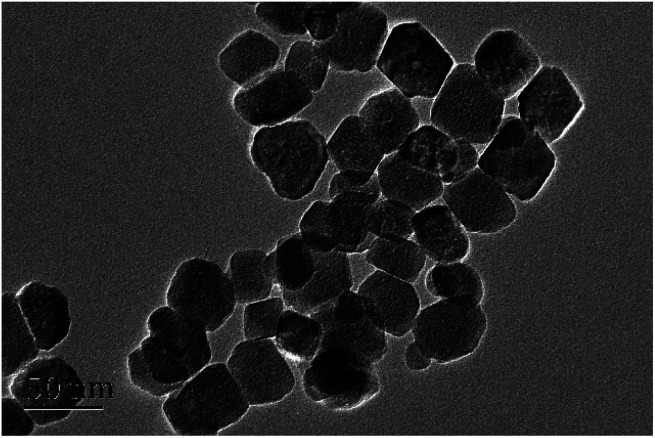
TEM of Fe_3_O_4_@PEI@PYBA.

Transmission electron microscopy (TEM) was used to investigate the morphology of the prepared MNPs. As depicted in Fig. 2, the obtained Fe_3_O_4_@PEI@PYBA exhibited good dispersibility and relatively homogeneous size distribution. The size of the Fe_3_O_4_@PEI@PYBA was about 50 nm at diameter, which implied large specific surface area.

### Selectivity

**Fig. 3:**
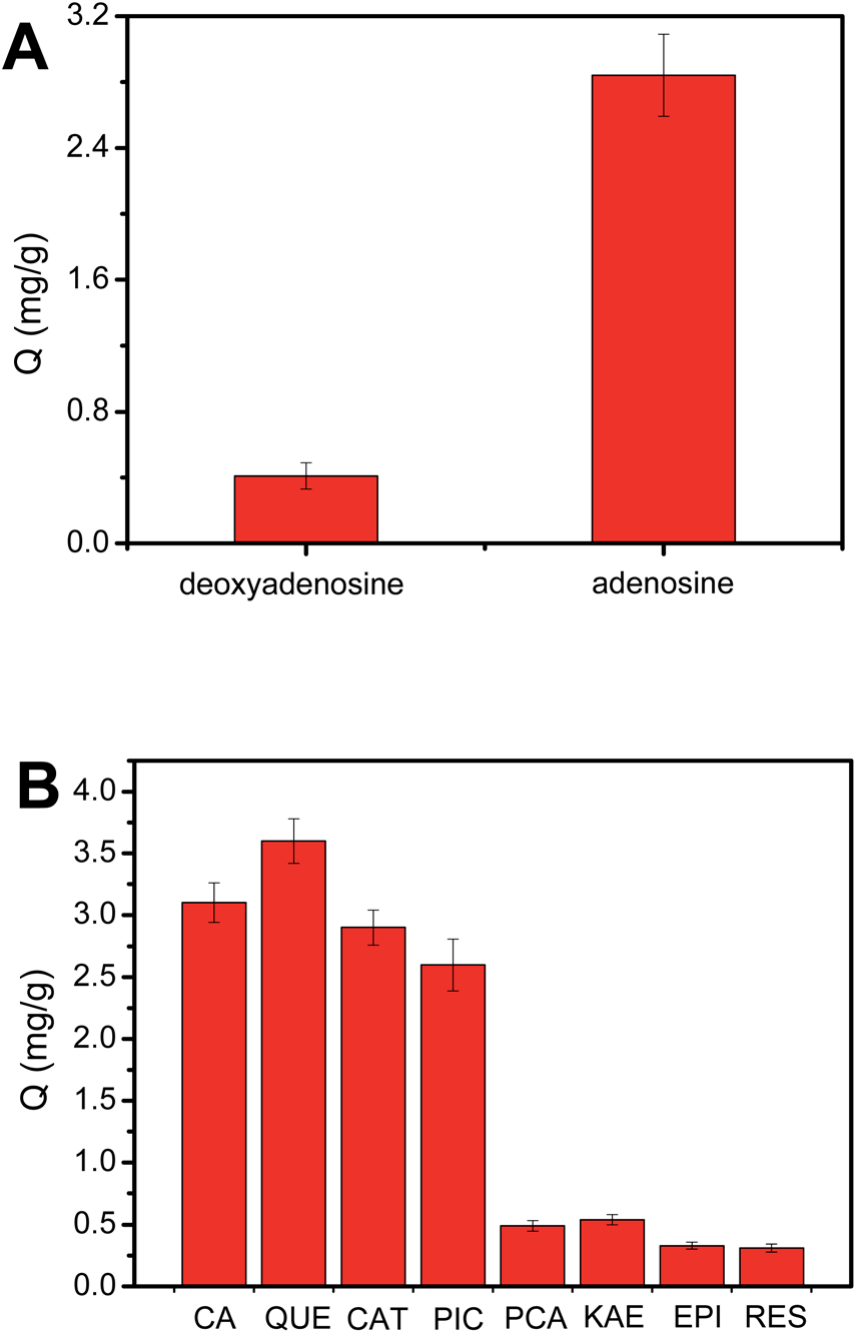
The binding capacity of Fe_3_O_4_@PEI@PYBA for different samples. Binding buffer: 50 mM sodium phosphate buffer (pH 7.0); elution solution: 100 mM HAc (pH 2.7); samples: (A) 0.50 mg mL^−1^ adenosine or deoxyadenosine; (B) 0.50 mg mL^−1^ CA, QUE, CAT, PIC, PCA, KAE, EPI or RES dissolved in binding buffer.

In order to investigate the selectivity of the obtained Fe_3_O_4_@PEI@PYBA, adenosine and deoxyadenosine were used as a *cis*-diol-containing sample and a non-*cis*-diol sample, respectively. As shown in Fig. 3A, Fe_3_O_4_@PEI@PYBA exhibited higher binding capacity with adenosine than that with deoxyadenosine, which indicated excellent selectivity. To further demonstrate the selectivity of Fe_3_O_4_@PEI@PYBA toward *cis*-diol-containing polyphenols, CA, QUE, CAT and PIC were used as *cis*-diol-containing polyphenols and PCA, KAE, EPI and RES were used as non-*cis*-diol analogue. It can be observed from Fig. 3B that *cis*-diols polyphenols exhibited higher binding capacity than non-*cis*-diol polyphenols for Fe_3_O_4_@PEI@PYBA, indicating good selectivity.

### Optimization of the molecular weight of PEI

**Fig. 4:**
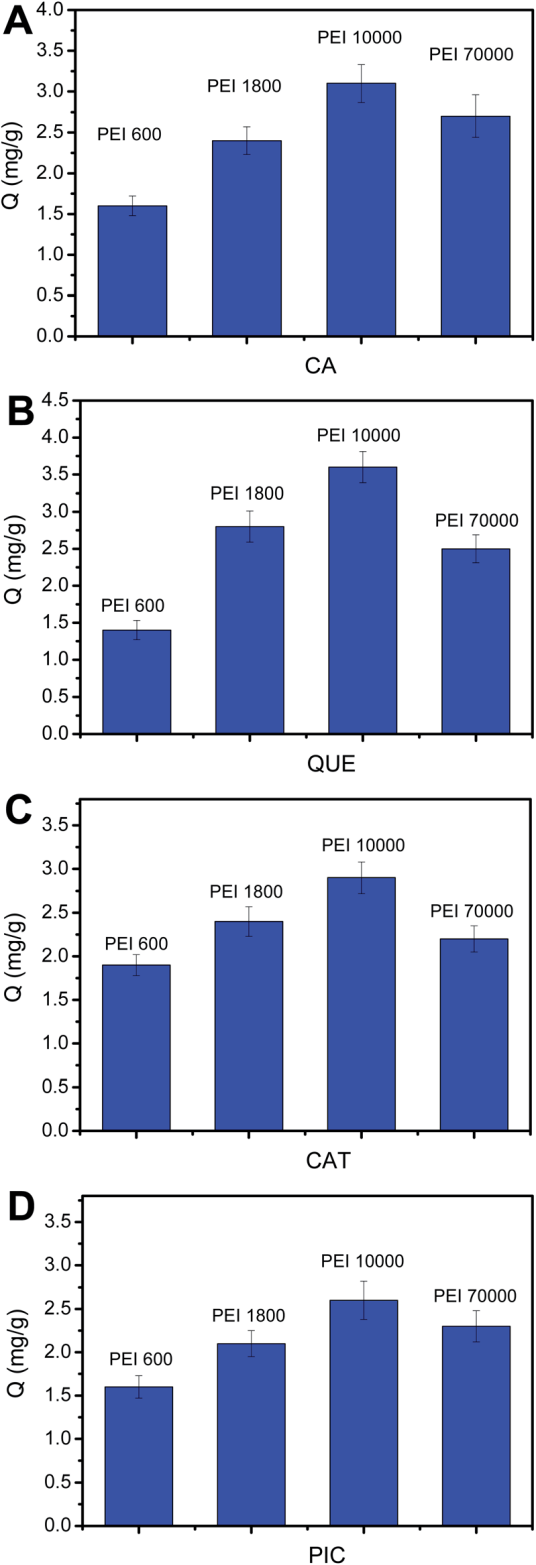
The binding capacity of CA (A), QUE (B), CAT (C) and PIC (D) samples captured by Fe_3_O_4_@PEI@PYBA through changing molecular weight of PEI. Binding buffer: 50 mM sodium phosphate buffer (pH 7.0); elution solution: 100 mM HAc (pH 2.7); the concentration of samples: 0.50 mg mL^−1^.

Generally, amount of the amine groups of PEI is proportional to the molecular weight of PEI. However, when molecular weight of PEI reaches a certain value, crosslinking reaction would occur and thereby a portion of amine groups may become invalid. Thus, it is necessary to investigate the effect of the molecular weight of PEI on the number of the binding sites, which can be reflected by binding capacity (*Q*, mg g^−1^). Based on this, we investigated the effect of the molecular mass of PEI on the binding capacity. It has been noted that the amount of functional monomer must be guaranteed to be excess. As depicted in Fig. 4, for every polyphenol, the binding capacity increased with the increase of molecular weight of PEI from 600 to 10 000. However, when molecular weight of PEI reaches 70 000, the binding capacity becomes lower. Thus, the PEI 10 000-modified PYBA-functionalized MNPs were considered as the optimal boronic acid-functionalized magnetic nanoparticles for further investigations and application.

### Determination of *Q*_max_ and *K*_d_

Binding capacity is a crucial factor in boronate affinity materials, which determines how high amount of targets would be captured by the affinity material. Binding affinity is one important binding property, which determines how low concentrations of *cis*-diol-containing polyphenols that Fe_3_O_4_@PEI@PYBA can enrich. Thus, it is quite important to evaluate the binding capacity and binding affinity. The maximum binding capacity (*Q*_max_) and dissociation constant (*K*_d_) of Fe_3_O_4_@PEI@PYBA with CA were investigated using UV-vis spectrophotometry.

**Fig. 5:**
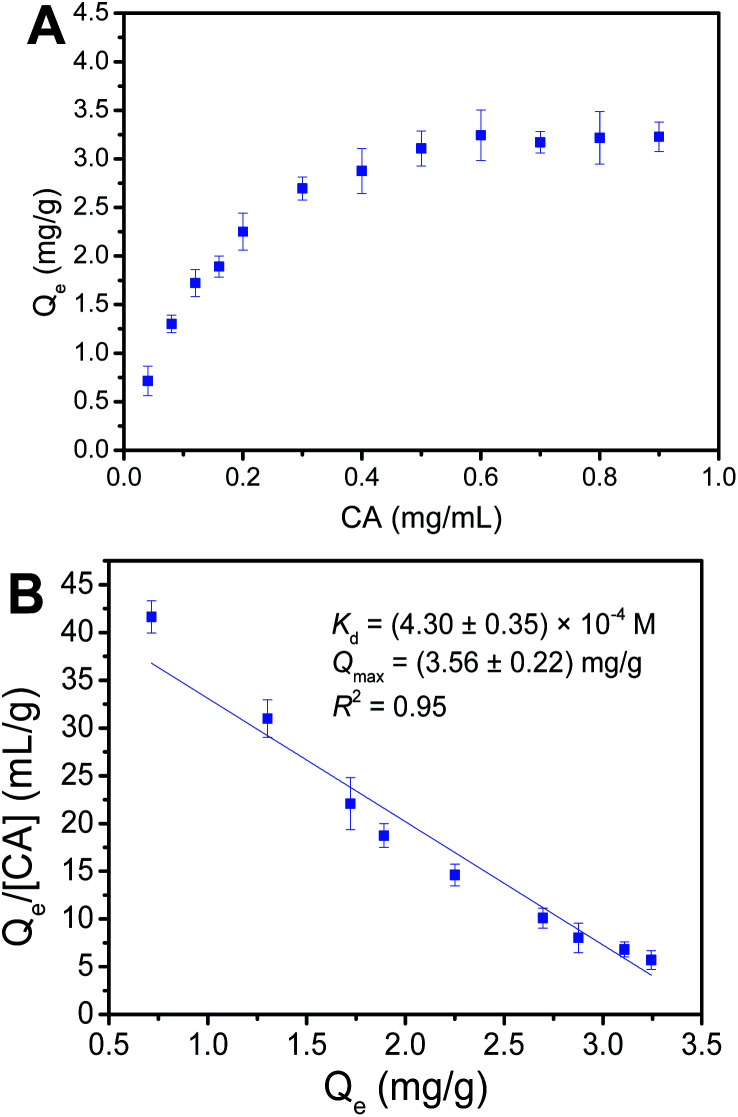
Binding isotherms (A) and Scatchard plots (B) for the binding of Fe_3_O_4_@PEI@PYBA with caffeic acid (CA).

As shown in Fig. 5, according to binding isotherms and Scatchard plots analyses, *Q*_max_ and *K*_d_ values of Fe_3_O_4_@PEI@PYBA were calculated to be (3.56 ± 0.22) mg g^−1^ and (4.30 ± 0.35) × 10^−4^ M, respectively. Clearly, the obtained binding capacity value was a little higher than that the previous boronate affinity materials provided.^39,40^ Thus, the amplified 6-aminopyridine-3-boronic acid moieties by using PEI is advantageous for the binding affinity and binding capacity of Fe_3_O_4_@PEI@PYBA.

### Adsorption time

**Fig. 6:**
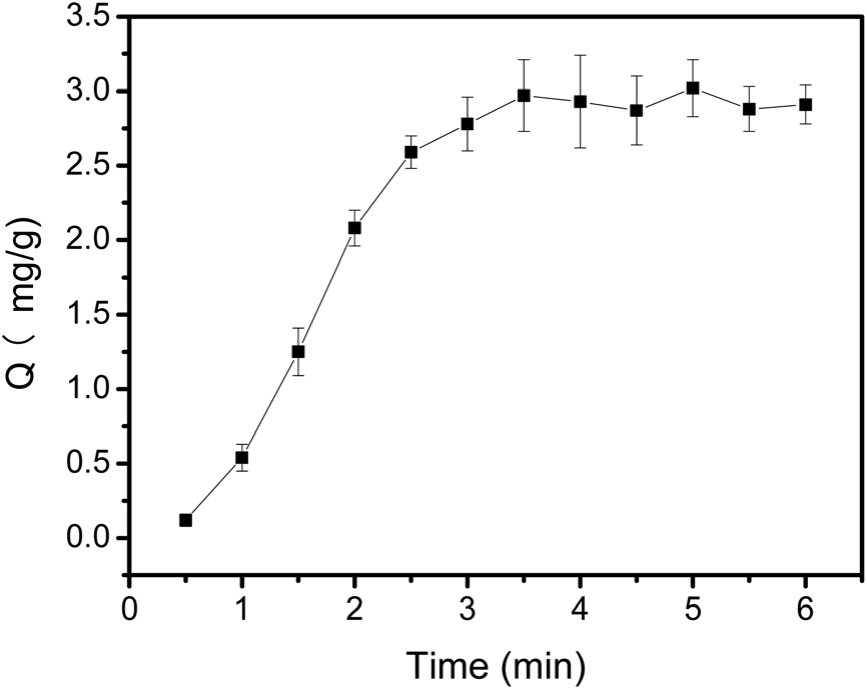
Binding equilibrium of Fe_3_O_4_@PEI@PYBA with CA. Sample: 0.50 mg mL^−1^ CA containing 50 mM phosphate, pH 7.0.

An appropriate adsorption time is required to obtain the highest binding capacity during the extraction procedure. To this end, the effect of extraction time on the binding capacity of polyphenols was investigated by varying time from 1 min to 8 min (Fig. 6). It can be observed from Fig. 6, the binding capacity of Fe_3_O_4_@PEI@PYBA for CA exhibited a fast adsorption rate within the first 3 min. After 3 min, the binding capacity nearly kept essentially constant. This possible explanation is that amount of binding sites at the beginning made CA be easily bound to Fe_3_O_4_@PEI@PYBA and the binding reaction would reach equilibrium at last. The results indicated that most of the binding sites had been occupied by CA in 3 min situation, which was compared to that PEI-assisted 3-carboxybenzoboroxole functionalized MNPs exhibited.^39^ This adsorption time at chemical equilibrium for Fe_3_O_4_@PEI@PYBA is much shorter than that for other boronate affinity materials (30–90 min).^44–48^ This may result from its high binding force of polyphenols during the extraction. In the light of these results, 3 min was chosen as the optimum adsorption time for extraction.

### Effect of competing saccharides on the extraction of polyphenols by Fe_3_O_4_@PEI@PYBA

**Fig. 7:**
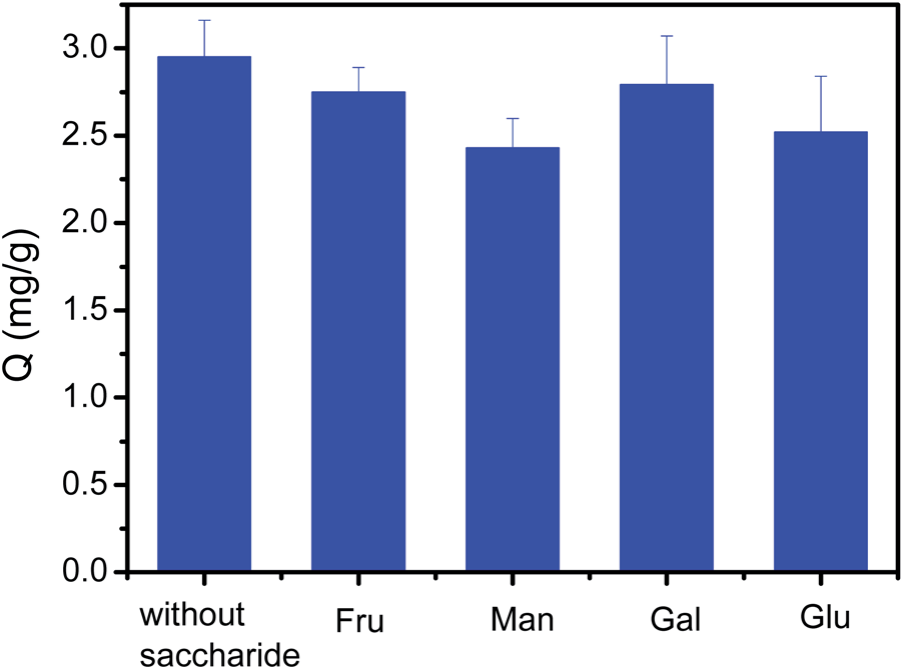
Effect of competing saccharides on the binding capacity of Fe_3_O_4_@PEI@PYBA for CA. Samples: 0.1 mg mL^−1^ CA without or with 1 mg mL^−1^ saccharide, fructose (Fru), mannose (Man), galactose (Gal) and glucose (Glu).

Sugars is also a kind of *cis*-diol-containing compounds. Sugars and polyphenols co-exists in many actual samples such as in fruits, vegetables, tea, honey and coffee. The presence of sugars may greatly affect the binding capacity of Fe_3_O_4_@PEI@PYBA for polyphenols. Thus, the investigation on their competing binding with Fe_3_O_4_@PEI@PYBA is necessary. As depicted in Fig. 7, the binding capacity of Fe_3_O_4_@PEI@PYBA for CA in the presence of a competing monosaccharide at 10-fold higher concentration was only a little less than that in the absence of sugar. Clearly, the presence of the competing saccharide has no apparent effect on the binding of CA to Fe_3_O_4_@PEI@PYBA. This possible explanation is that the Fe_3_O_4_@PEI@PYBA exhibited much higher binding affinity towards polyphenols than towards saccharides because polyphenols contain phenols while saccharides contain aliphatic alcohol. Thus, the Fe_3_O_4_@PEI@PYBA was very tolerant of the interference of competing saccharides.

### Binding pH

**Fig. 8:**
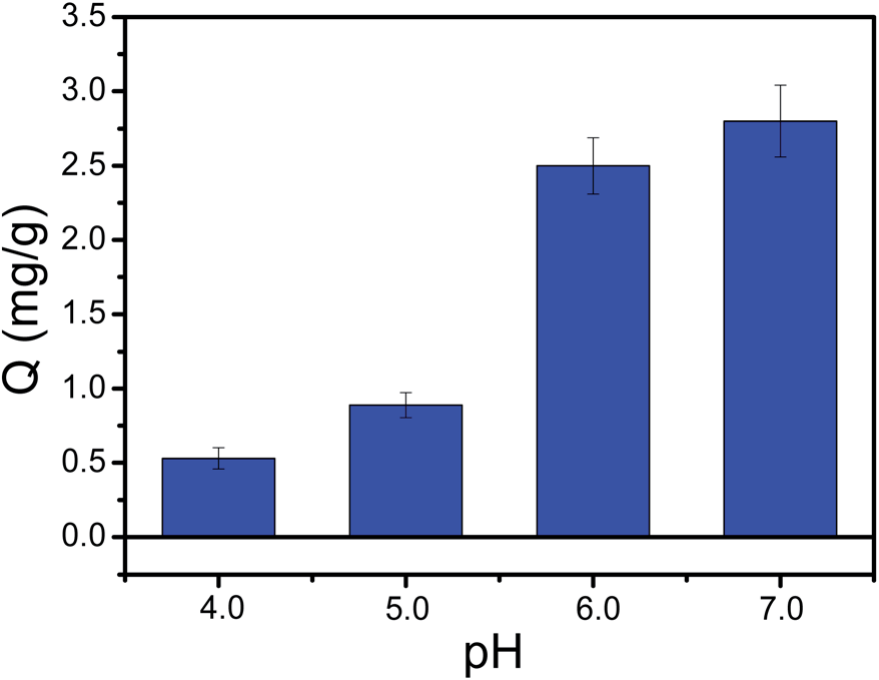
pH on the effect of the binding capacity of Fe_3_O_4_@PEI@PYBA towards CA. Binding buffer: 50 mM sodium phosphate buffer at pH 4.0–7.0.

Binding pH value is an important binding property of boronic acid-functionalized materials. As we know, conventional boronate affinity materials used boronic acid with high p*K*_a_ value as affinity ligand, which requires a basic pH condition. That's to say, the binding affinity is higher at alkaline condition than neutral or acidic condition. Unfortunately, the basic pH condition is easy to oxidize the polyphenols. It is quite necessary to prepare boronic acid-functionalized materials that can work at neutral or acidic condition. To address this issue, PYBA with electron-withdrawing group can be used to reduce the binding pH to neutral or acidic conditions. Thus, the prepared PYBA-functionalized MNPs can provide a lower binding pH value. As depicted in Fig. 8, the Fe_3_O_4_@PEI@PYBA NPs exhibited high binding capacity for CA at pH ≥ 6.0. The Fe_3_O_4_@PEI@PYBA are still able to well capture CA even at pH 4.0 and 5.0, which was superior to PEI-assisted 3-carboxybenzoboroxole functionalized MNPs.^39^ The above results implied that Fe_3_O_4_@PEI@PYBA can work under neutral or acidic conditions.

### Reproducibility and re-usability

In order to investigate reproducibility of the obtained Fe_3_O_4_@PEI@PYBA, the prepared six batches of Fe_3_O_4_@PEI@PYBA on different days were applied and the measurements replicated three times in parallel. By calculation, the average *Q* of the total Fe_3_O_4_@PEI@PYBA for CA was 2.82 mg g^−1^, and the relative standard deviations (RSD) were less than 7.3% for CA, which indicated that the reproducibility of the Fe_3_O_4_@PEI@PYBA was satisfactory.

Re-usability is one of the most important properties of boronate affinity materials in practical application. As far as we know, the Fe_3_O_4_@PEI@PYBA can be reused after washing with acid solution. In order to investigate the re-usability of Fe_3_O_4_@PEI@PYBA, the adsorption–regeneration cycle was repeated ten times with the same Fe_3_O_4_@PEI@PYBA. The results indicated that Fe_3_O_4_@PEI@PYBA are very stable and adsorption capacity still maintain 91.4% of the first cycle after five adsorption–desorption cycles. That's, the Fe_3_O_4_@PEI@PYBA could be well reused at least five times.

## Conclusions

In this study, the PEI-assisted boronic acid-functionalized MNPs were prepared for the selective enrichment of polyphenols. By using branched PEI as the scaffold for amplification of boronic acid sites and 6-aminopyridine-3-boronic acid as the affinity ligand, PEI-assisted boronic acid-functionalized MNPs can obtain high binding capacity. The obtained dissociation constant is 10^−4^ M. In addition, the combination of PEI-assisted synergistic effect and novel boronic acid ligands can lead to lower binding pH. Due to the high binding strength, high binding capacity and the low binding pH, the boronate affinity MNPs can be directly applied to selective enrichment of polyphenols in real samples.

## Conflicts of interest

There are no conflicts to declare.

## Supplementary Material
